# Effects of exercise on capillaries in the white matter of transgenic AD mice

**DOI:** 10.18632/oncotarget.19505

**Published:** 2017-07-22

**Authors:** Yi Zhang, Feng-Lei Chao, Chun-Ni Zhou, Lin Jiang, Lei Zhang, Lin-Mu Chen, Yan-Min Luo, Qian Xiao, Yong Tang

**Affiliations:** ^1^ Department of Laboratory Medicine, Key Laboratory of Diagnostic Medicine, Ministry of Education, Chongqing Medical University, Chongqing 400016, PR China; ^2^ Department of Histology and Embryology, Chongqing Medical University, Chongqing 400016, PR China; ^3^ Laboratory of Stem Cells and Tissue Engineering, Chongqing Medical University, Chongqing 400016, PR China

**Keywords:** capillary, white matter, Alzheimer’s disease, running exercise, stereology

## Abstract

Previous studies have shown that exercise can prevent white matter atrophy in APP/PS1 transgenic Alzheimer’s disease (AD) mice. However, the mechanism of this protective effect remains unknown. To further understand this issue, we investigated the effects of exercise on the blood supply of white matter in transgenic AD mice. Six-month-old male APP/PS1 mice were randomly divided into a control group and a running group, and age-matched non-transgenic littermates were used as a wild-type control group. Mice in the running group ran on a treadmill at low intensity for four months. Then, spatial learning and memory abilities, white matter and white matter capillaries were examined in all mice. The 10-month-old AD mice exhibited deficits in cognitive function, and 4 months of exercise improved these deficits. The white matter volume and the total length, total volume and total surface area of the white matter capillaries were decreased in the 10-month-old AD mice, and 4 months of exercise dramatically delayed the changes in these parameters in the AD mice. Our results demonstrate that even low-intensity running exercise can improve spatial learning and memory abilities, delay white matter atrophy and protect white matter capillaries in early-stage AD mice. Protecting capillaries might be an important structural basis for the exercise-induced protection of the structural integrity of white matter in AD.

## INTRODUCTION

Alzheimer's disease (AD) is a neurodegenerative disease characterized by progressive cognitive and memory dysfunction [1]. The impact of serious intellectual disability on AD patients and the burden of a low quality of life on families have led AD to become a serious social problem. Studies of AD pathogenesis and early-stage AD prevention are urgently needed [2]. Recently, increasing numbers of studies have reported a large variety of vascular changes in the AD brain, and these changes could lead to brain capillary microcirculation changes and hypoperfusion, which might be a critical cause of the cognitive decline associated with AD [3–5]. Previous studies have reported that spiral-like vascular structures, resulting in vascular tortuosity and occlusion, are often associated with white matter lesions in AD [6]. Moreover, compared with other brain regions, white matter has a lower density of capillaries and is more susceptible to hypoxic-ischemic injury [7, 8]. Research has suggested that in early-stage AD, damage to the structural integrity of white matter is closely related to cognitive decline [9–13]. Therefore, researchers have speculated that capillary changes within white matter might be a crucial cause of cognitive decline in AD and a potential target for AD treatment [14, 15]. However, few studies have investigated capillary changes within the white matter of early-stage AD patients or AD mice using accurate three-dimensional quantitative methods.

Despite the extensive efforts of researchers worldwide, there is no effective cure for this devastating disease, and researchers have been searching for effective preventive strategies to delay the progression of early-stage AD. Clinical studies have suggested that physical exercise is a simple and economical lifestyle factor that might delay AD progression [16]. Animal studies have shown the same effect. Cotman reported that in transgenic AD mice, early intervention in the form of running exercise could delay the rate of cognitive decline [17]. However, the mechanisms of the protective effect of physical exercise against AD remain inconclusive. The main function of running exercise is to improve blood circulation and increase cerebral perfusion and blood oxygen content [18]. Cerebral blood vessels and capillaries might be crucial mechanistic components of the protective effect of exercise against AD [19–22]. Researchers have reported that running exercise can increase the levels of angiogenic factors and induce angiogenesis in the normal brain [23, 24]. Using stereological methods, our previous quantitative studies revealed that long-term running exercise can protect the capillaries within the cortex of middle-aged and old rats [25, 26]. Moreover, our team found that running exercise could protect the structural integrity of white matter in APP/PS1 transgenic mice [13]. However, whether white matter capillaries might be an important structural basis for the exercise-induced protection of white matter in APP/PS1 transgenic mice remains unknown.

In this study, we used an accurate and quantitative three-dimensional stereological method to study the white matter and white matter capillaries of 10-month-old APP/PS1 mice. Previous studies have indicated that estrogen might play a key role in the decline of brain function with aging [27–30], and our previous study showed that estrogen could protect the white matter of ovariectomized middle-aged female rats [31]. To separate the influence of estrogen from the effects of exercise on the white matter capillaries of AD mice, we subjected 6-month-old male APP/PS1 mice to treadmill exercise for four months. After the transgenic APP/PS1 AD mice completed the treadmill exercise-based behavioral intervention, we estimated the effects of treadmill exercise on learning, memory, white matter and white matter capillaries.

## RESULTS

### Improved spatial learning and memory in exercised AD mice

**Figure 1:**
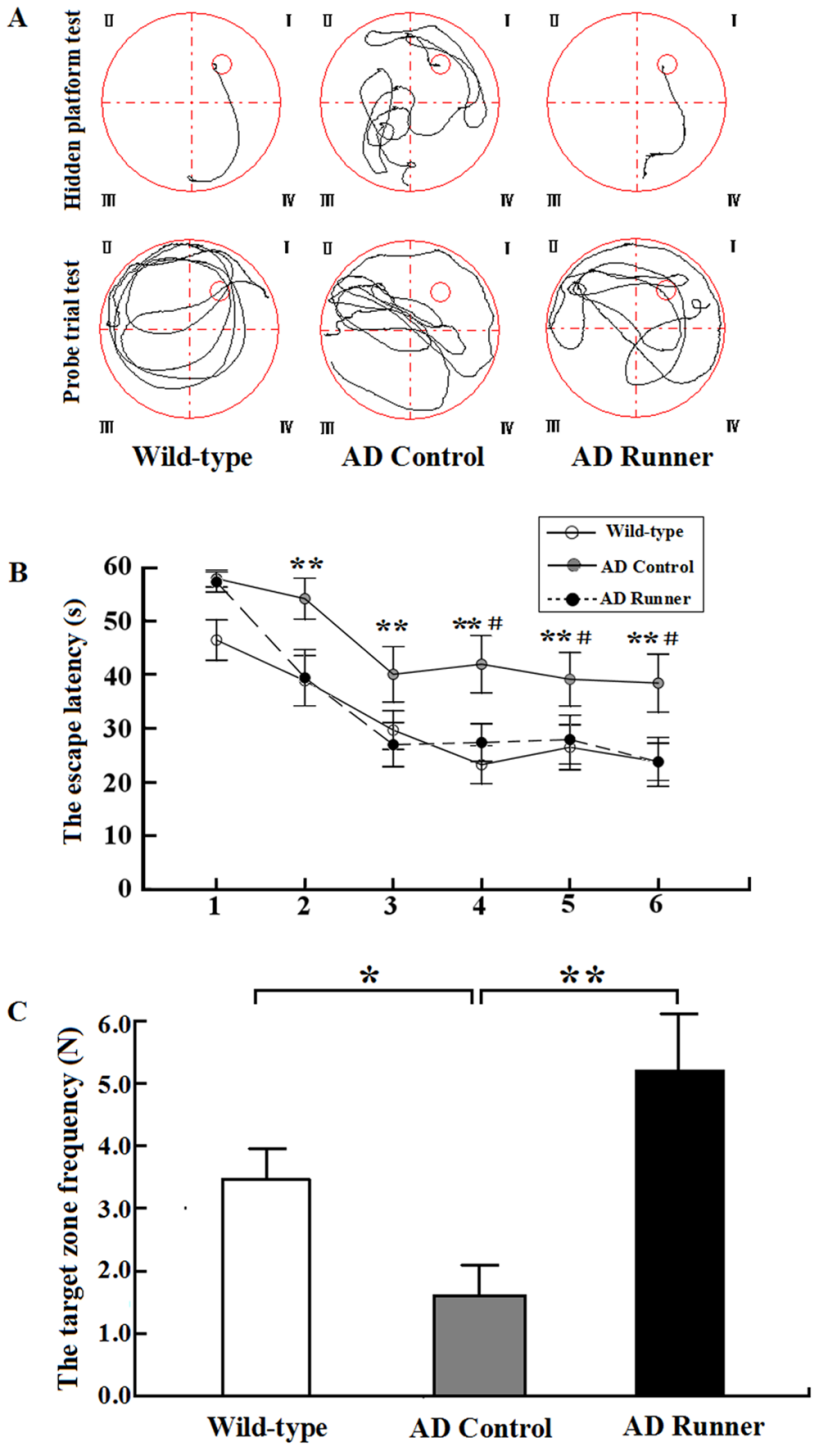
**(A)** The tracked locations of the Wild-type, APP/PS1 Control and APP/PS1 Runner mice in the hidden platform test. The Roman numerals (I, II, III, and IV) represent the first, second, third, and fourth quadrants, respectively, of the Morris water maze. **(B)** The escape latencies of the Wild-type, APP/PS1 Control and APP/PS1 Runner mice in the Morris water maze positioning navigation test. The 1,2,3,4,5 and 6 represent the latency day. Each point represents the average of four escape latencies (mean ± SEM). The asterisk (*) indicates the escape latency of the Wild-type mice compared with that of the APP/PS1 Control group in the Morris water maze positioning navigation test. The pound sign (^#^) indicates the escape latency of the APP/PS1 Runner mice compared with that of the APP/PS1 Control mice in the Morris water maze positioning navigation test. ** *p* < 0.01. ^#^*p* < 0.05. **(C)** The platform-crossing frequency of the Wild-type, APP/PS1 Control and APP/PS1 Runner mice (mean ± SEM) in the probe trial tests. * *p* < 0.05. ** *p* < 0.01.

Traces of the movement of mice during the hidden platform test and the probe trial test for the Wild-type, AD Control and AD Runner mice are presented in Figure 1A.

### Escape latency on the hidden platform test

Repeated-measures analysis of variance (ANOVA) was used to analyze the hidden platform task data. The results showed that there were significant differences in the hidden platform test among the three groups (F = 5.419; *p* = 0.012). The escape latency of the hidden platform test in the AD Control mice was significantly longer than that in the Wild-type mice (*p* = 0.004), and this significant difference began on the second day (*p* = 0.009; Figure 1B). The escape latency of the hidden platform test in the AD Runner mice was significantly shorter than that in the AD Control mice (*p* = 0.027), and this significant difference began on the fourth day (*p* = 0.029; Figure 1B). There was no significant difference in escape latency between the AD Runner and Wild-type mice *(p* = 0.591; Figure 1B).

### Platform-crossing frequency of the probe trial test

One-way ANOVA was used to analyze the platform-crossing frequency. The results showed that there were significant differences among the three groups (F = 7.495; *p* = 0.003). The platform-crossing frequency of the AD Control mice was significantly less than that of the Wild-type mice (*p* = 0.039; Figure 1C). The platform-crossing frequency of the AD Runner mice was significantly greater than that of the AD Control mice (*p* = 0.001; Figure 1C). There was no significant difference in platform-crossing frequency between the AD Runner and Wild-type mice (*p* = 0.054; Figure 1C).

### Total white matter volume

For the Wild-type group, 246 points touching white matter were counted per mouse hemisphere. For the AD Control group, 383 points touching white matter were counted per mouse hemisphere. For the AD Runner group, 375 points touching white were counted per mouse hemisphere. Since the brain sections of the three groups were imaged at different magnifications using an anatomy microscope, the area corresponding to each measuring point, a (p), varied among the three groups. In the Wild-type group, the area corresponding to each measuring point, a (p), was 0.183 mm^2^. The a (p) in the AD Control group was 0.087 mm^2^, and that of the AD Runner group was 0.111 mm^2^. The observed coefficient of variation (OCV) and observed coefficient of error (OCE) values are shown in Table 1.

**Table 1:** Stereological results of the Wild-type, AD Control and AD Runner groups of APP/PS1 transgenic mice

		*Vwm* (mm^3^)	*Lv (cap / wm)*	*L (cap, wm)* (m)	*Vv (cap / wm)*	*V (cap, wm)*(mm^3^)	*Sv (cap / wm)*	*S(cap, wm)* (mm^2^)
**Wild-type**	Mean	14.88	0.73	10.83	0.007	0.11	7.81	116.62
	SD	0.71	0.09	1.67	0.002	0.029	0.85	16.86
	OCV (%)	4.76	12.79	15.44	25.51	27.44	10.83	14.46
	OCE (%)	1.39	11.81	11.89	12.27	12.35	12.37	12.44
**AD Control**	Mean	11.11	0.35	3.90	0.003	0.04	4.61	51.08
	SD	1.05	0.06	0.48	0.001	0.009	0.28	4.52
	OCV (%)	9.42	17.21	12.21	27.71	24.42	6.02	8.86
	OCE (%)	1.23	18.22	18.26	18.54	18.58	18.73	18.77
**AD Runner**	Mean	13.88	0.55	7.65	0.006	0.09	6.84	95.01
	SD	0.83	1.00	1.75	0.001	0.023	1.09	17.75
	OCV (%)	5.97	19.12	22.94	21.37	25.08	15.91	18.68
	OCE (%)	1.07	14.47	14.51	13.33	13.37	15.43	15.47

* Vwm is the total volume of white matter, Lv(cap/wm)is the length density of the capillaries in the white matter, L(cap, wm) is the total length of the capillaries in the white matter, Vv(cap / wm) is the volume density of the capillaries in the white matter, V(cap, wm) is the total volume of the capillaries in the white matter, Sv(cap / wm) is the capillary surface area density in the white matter, and S(cap, wm) is the total surface area of the capillaries in the white matter. The mean value (Mean), standard deviation (SD), observed coefficient of variation (OCV) and observed coefficient of error (OCE) of each parameter are provided.

**Figure 2:**
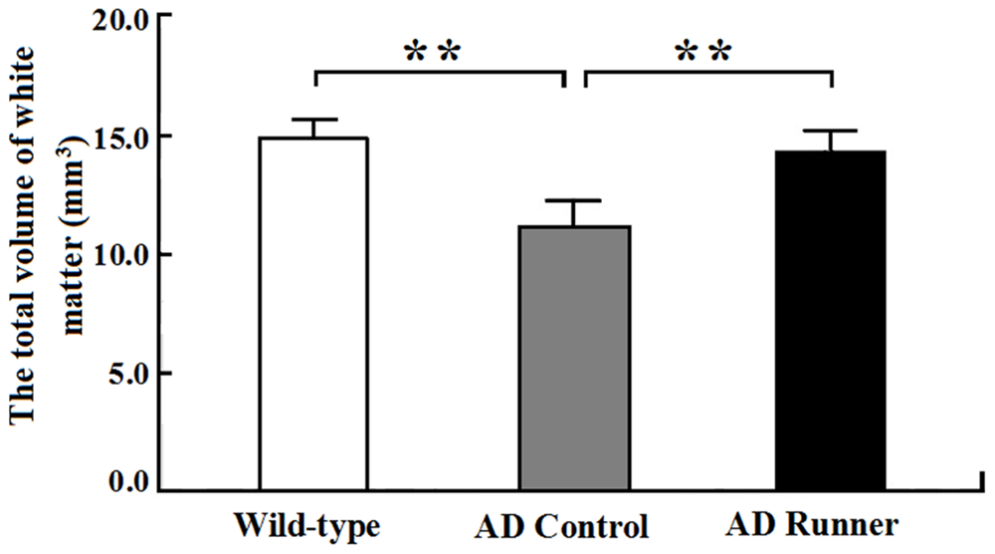
The total white matter volume of the Wild-type, APP/PS1 Control and APP/PS1 Runner mice (mean ± SD) ** *p* < 0.01.

The total white matter volume of the Wild-type, AD Control, and AD Runner mice was 14.88 ± 0.71 mm^3^, 11.11 ± 1.05 mm^3^, and 13.88 ± 0.83 mm^3^, respectively. One-way ANOVA was used to analyze the total white matter volume among the three groups. The results showed that there were significant differences among the three groups (F = 30.126; *p* = 0.000). The white matter volume of the AD Control mice was significantly smaller than that of the Wild-type mice (*p* = 0.000) (Figure 2). The white matter volume of the AD Runner mice was greater than that of the AD Control mice (*p* = 0.000) (Figure 2). There was no significant difference in white matter volume between the AD Runner and Wild-type mice (*p* = 0.064) (Figure 2).

### White matter capillaries

**Figure 3:**
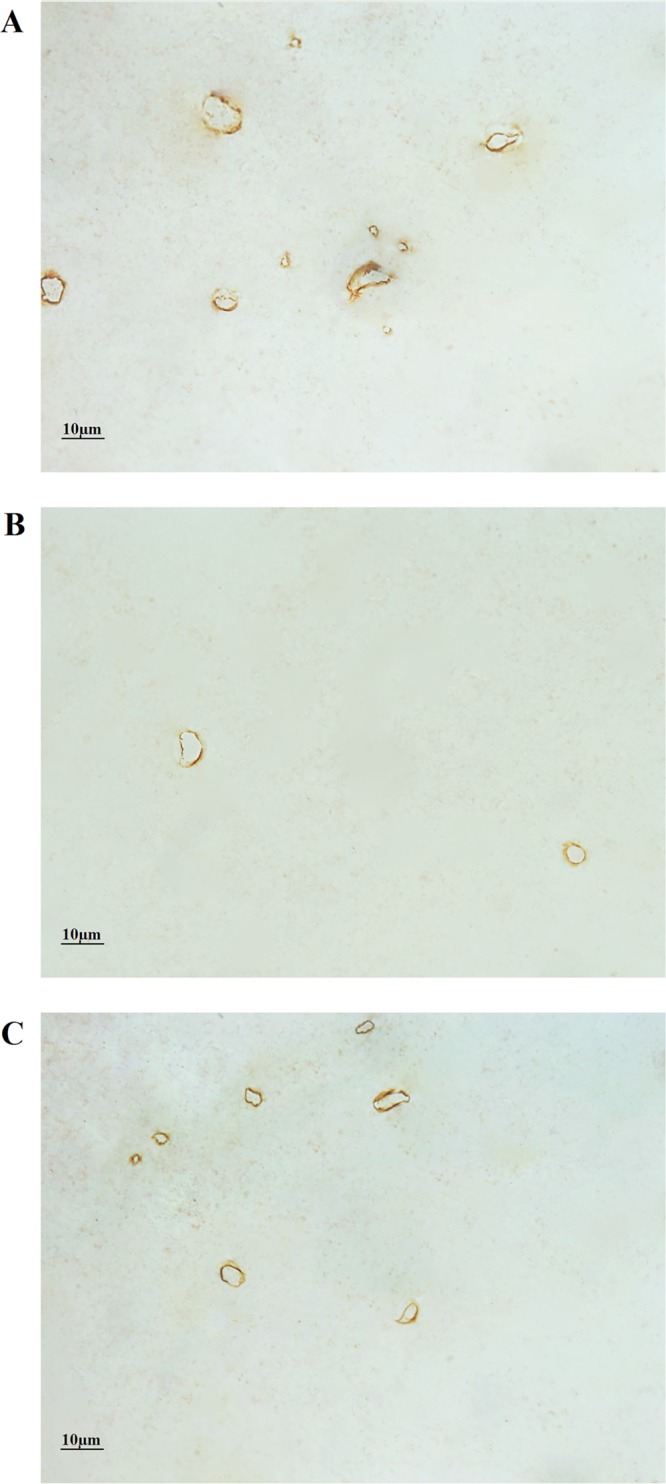
**(A)** The white matter capillaries of the 10-month-old Wild-type mice. **(B)** The white matter capillaries of the 10-month-old APP/PS1 Control mice. **(C)** The white matter capillaries of the 10-month-old APP/PS1 Runner mice. Bar = 10 μm.

The results of the immunohistochemistry-based morphological examination of the white matter capillaries of the Wild-type, AD Control and AD Runner mice are shown in Figure 3.

For the Wild-type group, on average, 254 images of white matter were randomly captured per mouse; 894 capillary profiles, 1390 points touching capillaries and 643 intersections between the test lines and capillaries in the white matter were counted per mouse hemisphere. For the AD Control group, on average, 267 images from the white matter were randomly captured per mouse; 364 capillary profiles, 630 points hitting touching capillaries and 271 intersections between the test lines and capillaries in the white matter were counted per mouse hemisphere. For the AD Runner group, on average, 241 images from the white matter were randomly captured per mouse; 554 capillary profiles, 1162 points touching capillaries and 472 intersections between the test lines and capillaries in the white matter were counted per mouse hemisphere. The OCV and OCE values are shown in Table 1.

### Total length of white matter capillaries

**Figure 4:**
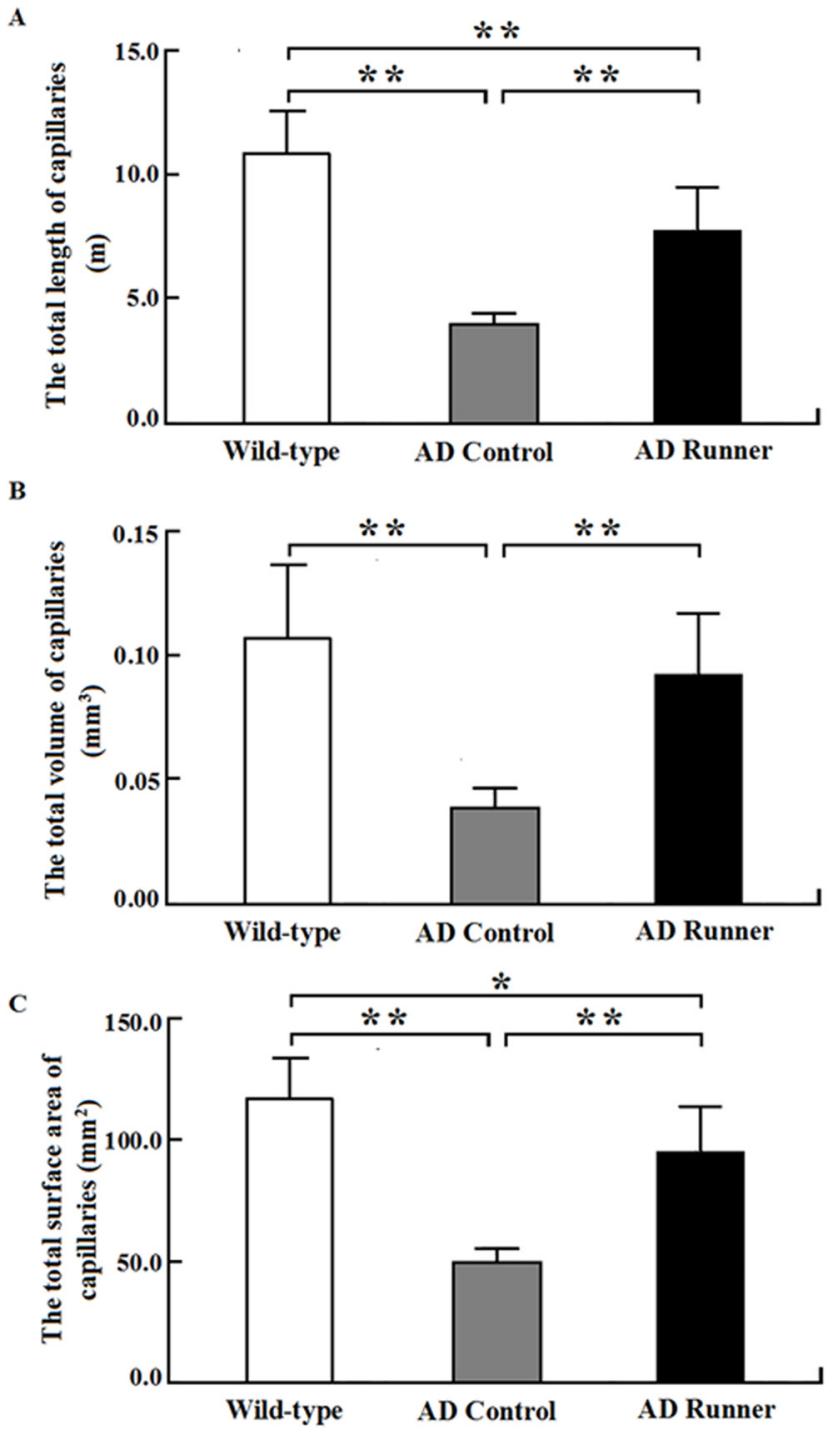
**(A)** The total white matter capillary length of the Wild-type, APP/PS1 Control and APP/PS1 Runner mice (mean ± SD). ** *p* < 0.01. **(B)** The total white matter capillary volume of the Wild-type, APP/PS1 Control and APP/PS1 Runner mice (mean ± SD). * *p* < 0.05, ** *p* < 0.01. **(C)** The total white matter capillary surface area of the Wild-type, APP/PS1 Control and APP/PS1 Runner mice (mean ± SD). ** *p* < 0.01.

The total length of white matter capillaries in the Wild-type, AD Control, and AD Runner mice was 10.83 ± 1.67 m, 3.90 ± 0.47 m, and 7.65 ± 1.75 m, respectively. One-way ANOVA was used to analyze the total length of white matter capillaries in the three groups. The results showed that there were significant differences among the three groups (F = 35.484; *p* = 0.000). The total length of white matter capillaries in the AD Control mice was significantly shorter than that in the Wild-type mice (*p* = 0.000; Figure 4A). The total length of white matter capillaries in the AD Runner mice was significantly longer than that in the AD Control mice (*p* = 0.002; Figure 4A). The total length of white matter capillaries in the AD Runner mice was significantly shorter than that in the Wild-type mice (*p* = 0.000; Figure 4A).

### Total volume of white matter capillaries

The total volume of white matter capillaries in the Wild-type, AD Control, and AD Runner mice was 0.1062 ± 0.0291 mm^3^, 0.0364 ± 0.0088 mm^3^, and 0.0924 ± 0.0232 mm^3^, respectively. One-way ANOVA was used to analyze the total volume of white matter capillaries in three groups. The results showed that there were significant differences among the three groups (F = 16.814; *p* = 0.000). The total volume of white matter capillaries in the AD Control mice was significantly smaller than that in the Wild-type mice (*p* = 0.000; Figure 4B). The total volume of white matter capillaries in the AD Runner mice was significantly greater than that in the AD Control mice (*p* = 0.000; Figure 4B). There was no significant difference in the total volume of white matter capillaries between the AD Runner and Wild-type mice (*p* = 0.296; Figure 4B).

### Total surface area of white matter capillaries

The total surface area of white matter capillaries in the Wild-type, AD Control, and AD Runner mice was 116.6 ± 16.8 mm^2^, 51.1 ± 4.5 mm^2^, and 95.0 ± 17.7 mm^2^, respectively. One-way ANOVA was used to analyze the total surface area of the capillaries in the white matter of three groups. The results showed that there were significant differences among the three groups (F = 32.407; *p* = 0.000). The total surface area of white matter capillaries in the AD Control and AD Runner mice was significantly smaller than that in the Wild-type mice (*p* = 0.000 and *p* = 0.020, respectively; Figure 4C). The total surface area of white matter capillaries in the AD Runner mice was significantly greater than that in the AD Control mice (*p* = 0.001; Figure 4C).

## DISCUSSION

AD is a neurodegenerative disease, and physical exercise is a simple and economical lifestyle factor to modify. A growing number of clinical studies have suggested that physical exercise might delay the onset and progression of AD [32–35]. Previous studies have found that wheel running exercise can prevent cognitive decline in transgenic AD mice, such as Tg2576 mice, APOE epsilon 4 mice and APP/PS1 double transgenic mice [36–38]. In our previous studies [13, 39] and the current study, we subjected APP/PS1 mice to a treadmill running exercise intervention from 6 to 10 months of age. The results showed that at 10 months, the Morris water maze performance of the AD Control mice in both the hidden platform task and the probe trial task was significantly worse than that of the Wild-type mice. After the running exercise intervention, the performance of the AD Runner mice was significantly better than that of the AD Control mice. Our results indicated that treadmill running exercise could improve spatial learning and memory in transgenic AD mice. These results were consistent with previous findings that treadmill running exercise could improve cognitive function in AD mouse models [40, 41]. Although running exercise is an effective intervention for AD, it is difficult for individuals to maintain a long-term running exercise regimen. In contrast, lower intensity exercise regimens are easier for individuals to maintain. Both Yuede et al. and Um et al. used 1 hour of treadmill running exercise per day, but in our study, we used only 20 min of treadmill running exercise per day, and no electric shock was used to force the mice to run. In addition, the speed of the treadmill running exercise was slower in the current study than in the studies of Yuede et al. and Um et al. Thus, the exercise intensity (10 m/min, 20 min/day) used in the current study was very low compared with that of other protocols reported in the literature. Our results indicated that low-intensity exercise also had positive effects on the cognitive function of AD mice.

Low-intensity running exercise had protective effects on the spatial learning and memory of AD mice. What effects might low-intensity exercise have on the neuroanatomy of AD mice? The hippocampus is widely considered the key neural structure related to spatial learning and memory. However, it is currently acknowledged that white matter is also involved in learning and memory [42]. In addition, the role of the brain’s white matter in active learning and memory may have been underestimated [43]. In recent years, accumulating evidence has shown that white matter lesions are closely related to cognitive decline in AD patients [9, 44, 45]. In addition, white matter lesions, rather than hippocampal atrophy, predict the incidence of AD [46]. Furthermore, AD animal model studies showed that axonal and myelin white matter injury was present before amyloid deposition and neurofibrillary tangle formation in gray matter [47]. The results of the current study showed that the white matter volume of the AD Control mice was significantly lower than that of the Wild-type mice. The current stereological results confirmed that the white matter changed with declined learning and memory in 10-month-old APP/PS1 mice. This converging evidence suggests that white matter atrophy might play an important role in the pathogenesis of AD-related cognitive impairment and might even affect the functionality of other brain regions and accelerate the cognitive decline associated with AD. Did running exercise have a beneficial effect on white matter atrophy in the AD brain? In this study, we used stereological methods to study the effects of running exercise on white matter in AD mice. The results showed that the total white matter volume was significantly greater in the AD Runner mice than in the AD Control mice, indicating that running exercise could delay white matter atrophy in AD mice. Previous studies have found that long-term running exercise can protect the structural integrity of white matter in physically fit older adults [23, 24]. In our previous study, we found that the myelinated fiber volume in the white matter of exercised AD mice was significantly greater than that of sedentary AD mice [13], which indicated that running exercise could delay the loss of myelinated fibers in the white matter of AD mice. AD pathogenesis is widely believed to be driven by the production and deposition of the β-amyloid peptide (Aβ). What might be the relation between the changes in white matter and the Aβ deposition in APP/PS1 AD mice? Although our current study did not investigate the effects of running exercise on Aβ deposition in the white matter of APP/PS1 AD mice, our team previously investigated this issue; we found Aβ plaque deposition in the white matter of 10-month-old APP/PS1 AD mice, but running exercise had no effect on Aβ plaque deposition in the white matter of APP/PS1 AD mice [13]. Along with our previous findings, the current results suggest that running exercise, even low-intensity running exercise, can protect the structural integrity of white matter in AD mice.

Running exercise can protect the structural integrity of white matter in AD mice. How did the running exercise regimen exert this protective effect on white matter in AD mice? Accumulating evidence from epidemiological and clinical studies suggests that vascular risk factors might be involved in the pathogenesis of AD [48–57]. Such risk factors might be related to Aβ accumulation in vessel walls, vascular fibrosis, and other ultrastructural changes in constituent endothelial and smooth muscle cells in AD [58]. Previous qualitative studies have described a large variety of microvascular changes (e.g., vessel looping, twining and braiding) in the AD brain [59]. Spiral-like vascular structures and periventricular venous collagenosis resulting in vascular tortuosity and occlusion are often associated with white matter lesions in AD [8]. In the current study, we used stereological methods to quantitatively investigate white matter capillaries in 10-month-old APP/PS1 mice. The results showed that the total length, volume and surface area of white matter capillaries were significantly less in 10-month-old APP/PS1 mice than in wild-type mice of the same age. Our data on the capillaries reflect the absolute values of capillaries in the white matter, thereby potentially avoiding problems related to the “reference space trap”. Moreover, white matter capillaries are not distributed randomly; this type of distribution may potentially result in a biased estimation. In our study, the isector technique was used to select isotropic, uniformly random sections such that all capillaries in three-dimensional space had an equal probability of being sampled [60]. Therefore, this study provided accurate, quantitative stereological research data on white matter capillaries in early-stage AD. Using stereological methods, Lee reported a 21% decrease in the number of capillaries in the corpus callosum in an AD mouse model, but no difference in the total capillary length [61]. There were several differences between this study by Lee et al. and our present study that might partly explain the discrepancy in the results, such as differences in the methods and in the age of the AD mice used. In fact, the number of capillaries in the region of interest could not be obtained without using a very complex stereological method. Ultimately, our present results clearly revealed a marked loss of capillaries within the white matter of APP/PS1 mice. White matter capillaries could potentially serve as an early target for the prevention and treatment of AD, which might encourage research to design new interventions, such as running exercise, which would target vascular risk factors at an earlier disease stage to prevent dementia or slow cognitive decline in early AD. Physical activity has been shown to up-regulate brain-derived neurotrophic factor, stimulate neurogenesis, and enhance neural plasticity and synaptic regeneration [17, 34, 35]. However, these changes are not directly associated with the benefits of exercise. We need to explore the main functions of running exercise in AD prevention, i.e., promoting brain blood circulation and capillary density. The main benefits of running exercise are improved blood circulation, increased cerebral perfusion and increased blood oxygen content [62]. In clinical studies, Ross et al. found that resistance exercise could increase the levels of the angiogenic factors VEGF-A, VEGF-C and VEGF-D [22], which suggests that running can promote the generation of capillaries. Additionally, Swain et al. [21] found that prolonged exercise induces angiogenesis in the rat brain and increases the cerebral blood volume of the primary motor cortex. Moreover, our previous quantitative study demonstrated that long-term running exercise can increase the capillaries within the cortices of middle-aged and old rats [25, 26]. What might be the mechanism underlying the exercise-induced benefits to capillaries within the rat cortex? Our previous study showed that endogenous nitric oxide could regulate capillaries in the cortex of Sprague Dawley rats via regulating blood vessel growth factors [63]. Is running exercise beneficial to white matter capillaries in the AD brain? In the present study, we used stereological methods to quantitatively study the effects of running exercise on white matter capillaries in transgenic AD mice. We found that the total length, volume and surface area of white matter capillaries in the AD Runner mice were significantly greater than those in the AD Control mice. The current findings indicate that running exercise, even low-intensity exercise such as that in the present study, has a protective effect on capillaries within the white matter of AD mice, which might be an important reason for the protective effect of exercise on the structural integrity of white matter in AD mice.

In conclusion, changes in the abundance of capillaries could be an important structural basis of white matter atrophy in AD mice. Low-intensity running exercise exerted a positive effect on not only learning and memory in APP/PS1 transgenic mice but also the structural integrity of their white matter and could delay changes in the abundance of capillaries within the white matter of the mice. This study shows that white matter capillaries could potentially serve as an early target for the prevention of AD and that running exercise might be an effective behavioral intervention for the early prevention of AD.

## MATERIALS AND METHODS

### Animals

**Figure 5:**
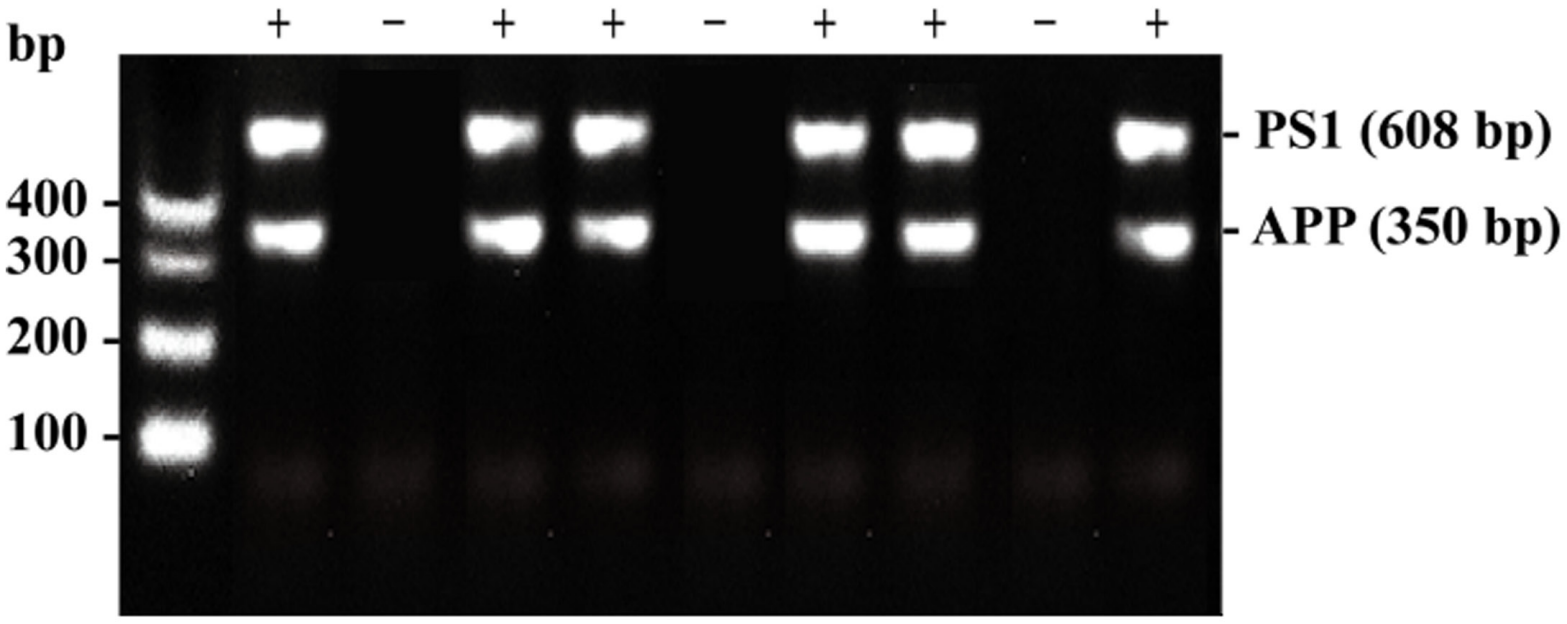
An illustration of the gene identification results obtained by genotyping The minus sign (-) indicates that this mouse expresses neither the APPswe gene nor the PSEN1 gene. The plus sign (+) indicates that this mouse expresses both the APPswe and PSEN1 genes.

The AD mouse strain used was B6C3-Tg(APPswe, PSEN1dE9)85Dbo/J, also known as APP/PS1. The primer sequences are shown below: Tg(APPswe) sequence: transgene = ∼ 350 bp. oIMR1597: 5’ GAC TGA CCA CTC GAC CAG GTT CTG 3’; oIMR1598: 5’ CTT GTA AGT TGG ATT CTC ATA TCC G 3’. Tg(PSEN1) sequence: transgene = ∼ 608 bp. oIMR1644: 5’ AAT AGA GAA CGG CAG GAG CA 3’; oIMR1645: 5’ GCC ATG AGG GCA CTA ATC AT 3’. An illustration of the gene identification is shown in Figure 5.

In our study, twenty 6-month-old male APP/PS1 mice and ten 6-month-old male non-transgenic littermate mice were randomly selected. All mice were provided by the Animal Model Institute of Nanjing University and bred in the Experimental Animal Center at Chongqing Medical University, PR China. The APP/PS1 transgenic AD mice were randomly divided into an APP/PS1 running group (AD Runner) and an APP/PS1 control group (AD Control); the wild-type littermate mice were used as the wild-type group (Wild-type); each group included ten mice. All mice were reared in a standard environment for 4 months. All animal care and treatment protocols conformed to the National Institutes of Health Guide for the Care and Use of Laboratory Animals (NIH Publication 85-23).

### Treadmill running exercise

The ten 6-month-old male APP/PS1 transgenic mice in the running group were placed on a treadmill to run regularly for four months (20 min per day, 5 days per week) [25, 39–41]. During the first two weeks, the running speed of 5 m/min was gradually increased to 10 m/min. For the rest of the experiment, the running speed was maintained at 10 m/min.

### Morris water maze

After the four months of running, the Morris water maze test was applied for seven consecutive days to evaluate spatial learning and memory in each group [64, 65]. The hidden platform test was conducted from the first day to the sixth day; the escape latency was recorded as a measure of spatial learning and memory. On the seventh day, the probe trial test was performed, in which the frequency of target zone crossings was recorded as a measure of spatial exploration. During the hidden platform test, the mouse was placed on the platform for 10 s of adaption prior to each trial. Then, the mouse was placed into the pool at a randomly chosen starting point in one of the four quadrants. The escape latency, or the time required for the mouse to find the platform location, was recorded for each quadrant. If the mouse did not reach the platform within 60 s, it was placed on the platform and allowed to stay there for 15 s. During the probe trial, the platform was removed. The two previously selected points that were the greatest distance from the platform were used as entry points. The frequency of target zone crossings during training was recorded.

### Tissue processing

**Figure 6:**
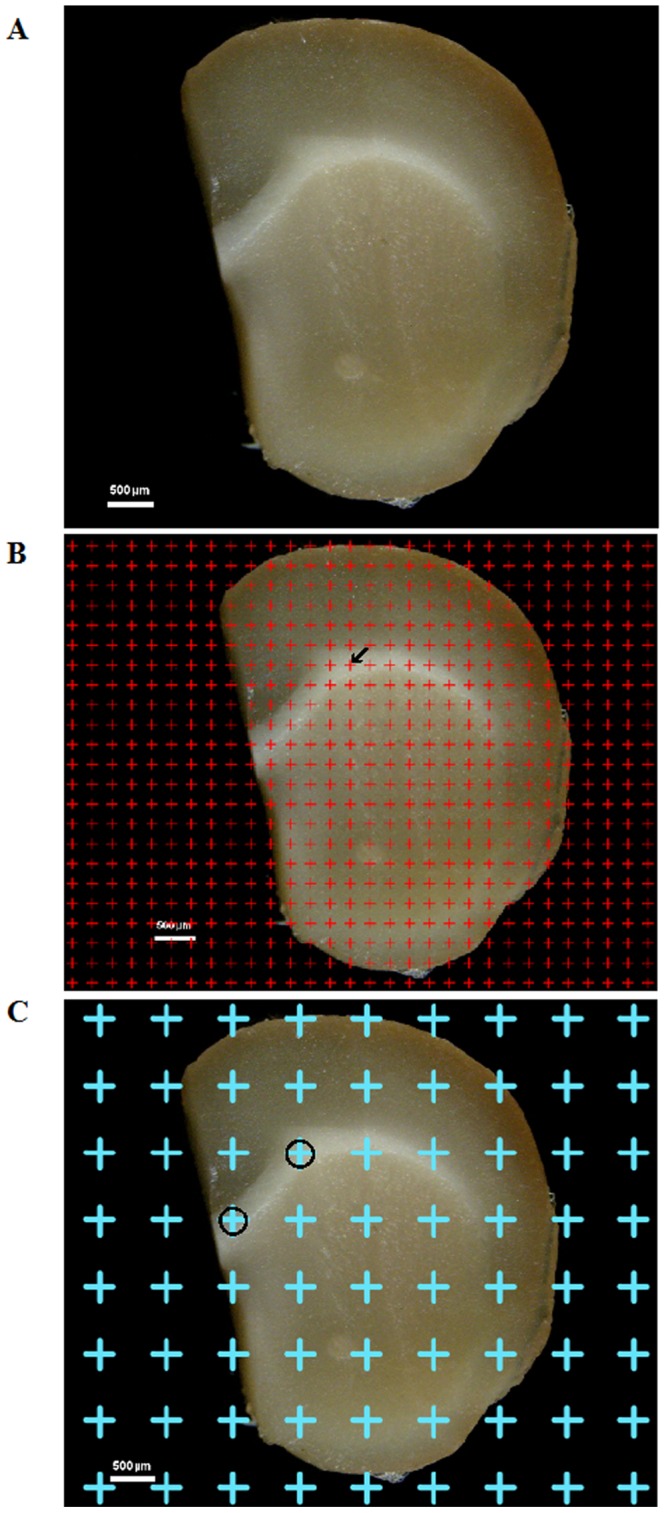
**(A)** One of the successive 1-mm-thick cerebral sections. Bar = 500 μm. **(B)** A point grid was randomly placed on one of the successive equidistant brain sections, and the points contacting white matter were counted. The arrow (←) indicates one of the counted points. Bar = 500 μm. **(C)** The point grid was randomly superimposed on the section, and the points contacting white matter were sampled. The circles (○) indicate the sampled white matter. Bar = 500 μm.

The mice were deeply anesthetized with 1% pentobarbital sodium (0.4 ml/100 g) and fixed via perfusion with 2% paraformaldehyde mixed with 2.5% glutaraldehyde. After the cerebral hemispheres were excised, they were embedded in 6% agar with the caudal surface facing down. Each hemisphere was sectioned into continuous 1-mm-thick coronal slices using mouse brain matrices (Figure 6A).

### Estimation of white matter volume

Images of the continuous 1-mm-thick coronal slices from the three groups were captured using the anatomy microscope. A transparent equidistant counting grid was randomly placed on the caudal surface of each 1-mm-thick section, and the points contacting white matter were counted (Figure 6B). The total white matter volume, *Vwm*, was calculated according to Cavalieri’s principle [66, 67]:

Vwm=t×a(p)×∑P(wm) (1)

where *Vwm* is the total white matter volume, a (*p*) is the area corresponding to each measuring point (0.59 mm^2^), t is the slice thickness (1 mm), and *ΣP (wm)* is the total number of counting points contacting white matter.

### White matter sampling and cryosectioning

The white matter tissue blocks (approximately 1 mm^3^) were randomly sampled based on the points that contacted the white matter (Figure 6C); five tissue blocks were sampled per mouse and were post-fixed in 4% paraformaldehyde for more than 2 hours. Then, the white matter samples were embedded in optimum cutting temperature (OCT) compound; the tissue blocks sampled were sectioned at 4 μm in parallel using a cryostat (LEICA CM1950, Germany). The process was repeated four times in different directions (5 sections were obtained per tissue block, and a total of 20 sections were obtained per mouse). Uniform, random and isotropic sections were obtained from each systematically randomly sampled block using the isector method [60]. The isector technique ensured that the capillaries of the white matter had the same probability of being sampled in each direction of three-dimensional space.

### Immunohistochemical procedures

Immunohistochemistry was performed using SPlink Detection Kits (SP 9002, ZSGB-BIO). The sections were fixed in acetone (4°C) for 10 min, and the antigens were retrieved in citrate buffer (0.01 M, pH 6.0, 99°C) for 10 min. Endogenous peroxidase activity was inhibited by incubating the sections with 3% hydrogen peroxide for 15 min, and nonspecific binding sites were blocked by incubating the sections with normal goat serum for 30 min at 37°C. Then, anti-mouse CD31 primary antibody (Abcam, UK) was added to the sections at a dilution of 1:500 in PBS [68]. The sections were incubated overnight at 4°C and then rewarmed at 37°C for 1 hour. The goat anti-mouse IgG secondary antibody solution was added, and the sections were incubated at 37°C for 30 min. Then, streptavidin-horseradish peroxidase (S-HRP) was added and incubated with the sections for 30 min. Subsequently, the sections were transferred to a diaminobenzidine (DAB) solution and immersed for approximately 1 min. Finally, the sections were dehydrated by sequential immersion in a gradient of ethanol solutions (70%, 80%, 90%, 100%, 100%, 100%; 10 min each) and cleared in xylene (3 × 10 min).

### Stereological sampling

The sections were viewed using a modified Olympus BX51 microscope (Olympus, Tokyo, Japan). A DP-70 video camera was mounted on top of the microscope and connected to a computer system. The boundaries of each section were drawn under low magnification (4×). Subsequently, images were captured under oil objective lens (100×) using the software of the stereology system (VIS). Vessels with a luminal diameter of less than 10 μm were defined as capillaries [69] and used for stereological analyses.

### Stereological analyses

#### Estimation of the total length of white matter capillaries

**Figure 7:**
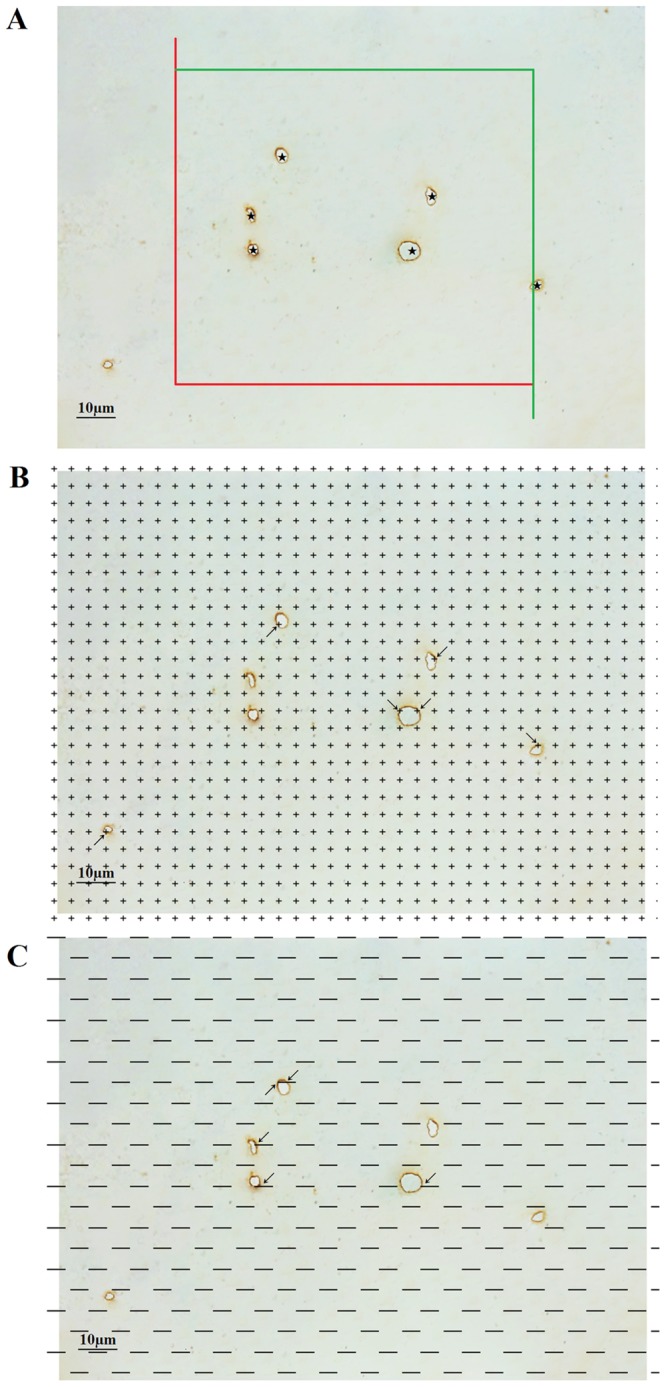
**(A)** An unbiased counting frame was randomly placed on the view of the white matter, and the capillary profiles inside the counting frame were counted, including those crossing the inclusion lines (green lines) but not crossing the exclusion lines (red lines). The stars (★) indicate counted capillaries. Bar = 10 μm. **(B)** A point grid was randomly placed on the view of the white matter, and the number of points contacting white matter and the number of points contacting capillaries were counted. The arrows (→) indicate the counted points. Bar = 10 μm. **(C)** Test lines were superimposed on the view of the white matter, and the number of intersections between the test lines and the capillary luminal surfaces was counted. The arrows (← & →) indicate the counted intersections between the test lines and the capillaries. Bar = 10 μm.

An unbiased counting frame was randomly superimposed onto the captured images [70]. The capillary profiles that were inside the counting frame or touching the top or right line (inclusion lines) were included in the count, and the capillary profiles that touched the left line, the bottom line or the extensions of the right or left line (exclusion lines) were excluded from the count (Figure 7A).

The length density of the capillaries in the white matter, *Lv(cap / wm)*, was calculated according to the following formula [66, 70, 71]:

$$Lv\left(cap/wm\right)=2\times \frac{\sum Q\left(cap\right)}{\sum A}$$ (2)

where *ΣQ(cap)* is the total number of capillary profiles counted per white matter section, and ΣA is the total area of the unbiased counting frames used when counting the white matter capillary profiles. Then, the total length of the white matter capillaries, *L(cap, wm),* was calculated according to the following formula:

L(cap,wm)=Lv(cap/wm)×Vwm (3)

#### Estimation of the total volume of white matter capillaries

A transparent equidistant counting grid was randomly placed on the randomly captured images. The test points contacting white matter capillaries and the test points contacting white matter were counted (Figure 7B). The volume density of white matter capillaries, *Vv(cap / wm)*, was calculated according to the following formula [67, 71]:

$$Vv\left(cap/wm\right)=\frac{\sum P\left(cap\right)}{\sum P\left(wm\right)}$$ (4)

where *ΣP(cap)* is the total number of counting points touching capillaries, and *ΣP (wm)* is the total number of counting points touching white matter. Then, the total volume of white matter capillaries, *V(cap, wm)*, was calculated according to the following formula:

$$V\left(cap,wm\right)=V_{v}\left(cap/wm\right)\times V_{wm}$$ (5)

#### Estimation of the total surface area of white matter capillaries

The test lines were randomly placed on the randomly captured images. The number of intersections between the test lines and the luminal surfaces of capillaries was counted, and the total length of test lines in the white matter was recorded (Figure 7C). The surface area density of white matter capillaries, *Sv(cap / wm)*, was calculated according to the following formula [67, 71]:

$$Sv\left(cap/wm\right)=2\times \frac{\sum I\left(cap\right)}{\sum L}$$ (6)

where *ΣI(cap)* is the total number of intersections between the test lines and capillaries, and *ΣL* is the total length of the test lines within white matter. Then, the total surface area of white matter capillaries, *S(cap / wm)*, was calculated according to the following formula:

S(cap,wm)=Sv(cap/wm)×Vwm (7)

### Statistics

The statistical analyses were performed using SPSS 19.0. The results of the Morris water maze test are presented as the mean ± standard error of the mean (SEM). The stereology results are presented as the mean ± standard deviation (SD). The Shapiro-Wilk test was used to evaluate whether the group means of all the data were normally distributed. Repeated-measures ANOVA was used to analyze the hidden platform task data, and one-way ANOVA was used to analyze the probe trial task data and the stereological data. The least significant difference (LSD) testwas used to detect the group differences in all parameters. A significance level of *p* < 0.05 was adopted throughout the analysis. Observed coefficient of variation (OCV) values and observed coefficient of error (OCE) values were calculated according to the method described by Gundersen et al. [72].
